# An Event Related Field Study of Rapid Grammatical Plasticity in Adult Second-Language Learners

**DOI:** 10.3389/fnhum.2017.00012

**Published:** 2017-01-24

**Authors:** Ainhoa Bastarrika, Douglas J. Davidson

**Affiliations:** ^1^Basque Center on Cognition, Brain and LanguageDonostia, Spain; ^2^Department of Linguistics and Basque Studies, University of the Basque CountryGasteiz, Spain

**Keywords:** grammatical plasticity, grammar learning, second language acquisition, MEG, Source analysis

## Abstract

The present study used magnetoencephalography (MEG) to investigate how Spanish adult learners of Basque respond to morphosyntactic violations after a short period of training on a small fragment of Basque grammar. Participants (*n* = 17) were exposed to violation and control phrases in three phases (pretest, training, generalization-test). In each phase participants listened to short Basque phrases and they judged whether they were correct or incorrect. During the pre-test and generalization-test, participants did not receive any feedback. During the training blocks feedback was provided after each response. We also ran two Spanish control blocks before and after training. We analyzed the event-related magnetic- field (ERF) recorded in response to a critical word during all three phases. In the pretest, classification was below chance and we found no electrophysiological differences between violation and control stimuli. Then participants were explicitly taught a Basque grammar rule. From the first training block participants were able to correctly classify control and violation stimuli and an evoked violation response was present. Although the timing of the electrophysiological responses matched participants' L1 effect, the effect size was smaller for L2 and the topographical distribution differed from the L1. While the L1 effect was bilaterally distributed on the auditory sensors, the L2 effect was present at right frontal sensors. During training blocks two and three, the violation-control effect size increased and the topography evolved to a more L1-like pattern. Moreover, this pattern was maintained in the generalization test. We conclude that rapid changes in neuronal responses can be observed in adult learners of a simple morphosyntactic rule, and that native-like responses can be achieved at least in small fragments of second language.

## Introduction

In research on second language acquisition (SLA), adult grammar learning has been characterized as a difficult or uncertain process (Weber-Fox and Neville, 1996), but work within the last decade has shown that for some groups of adult language learners, high levels of grammatical proficiency can be achieved in limited domains relatively quickly and effectively in focused learning tasks, and that there are also corresponding changes in the electrophysiological response that approximate those of adult first language (L1) responses in similar tasks (see Caffarra et al., 2015, for a recent review). Most previous studies have examined second language (L2) grammar learning using electroencephalography (EEG), however, and as a consequence, there is uncertainty in the literature about which brain areas correspond to these newly-emerging electrophysiological responses. Regarding magnetoencephalography (MEG) studies on the field, Davidson and Indefrey (2009b) examined grammar learning on adult learners of Dutch, the source reconstruction localized the evoked activity at left temporal and left inferior-frontal areas. However, participants were tested after 2 weeks and 3 months of formal course. In the present work we report a source reconstruction of evoked brain activity recorded using MEG before, during and after a few hours of grammar learning in adult Spanish [Spanish (SP), L1] learners of Basque [Basque (BQ), L2] with the goal of better characterizing the areas involved in the ability to recognize grammatical constraints.

Traditionally, research on SLA has focused on sensitive or potentially critical periods and the effects of different varieties of experience on SLA outcomes (Lenneberg, 1967; Birdsong, 2006). More recently there has been a shift in focus to characterizing how proficiency evolves over time during learning and development (Knudsen, 2004; Uylings, 2006; Zhang and Wang, 2007; Rodríguez-Fornells et al., 2009). Recent reviews have stressed that different language subsystems (e.g., semantics, syntactic, phonology) could rely on different cortical networks, and that they may have different sensitive period start- and end-points (Sanders et al., 2008). While there is now a database of areas implicated by bilingual functional Magnetic Resonance Imaging (fMRI) studies (e.g., Indefrey, 2006; Sebastian et al., 2011), to date there have been few comparisons of localized activity measured with electrophysiology. fMRI studies reviewed by Indefrey (2006) that focus on sentence comprehension and morpheme inflections have shown reliable differences during L1 and L2 processing, showing stronger involvement in left posterior areas in early stages of learning. In this study we used MEG and source reconstruction to uncover some of the broad areas that are involved in recognizing grammatical violations in learners.

Learning to use grammatical relations or rules in many cases involves learning to recognize dependencies that occur over time as words of a sentence are understood or produced. Morphosyntactic agreement relations such as gender, person or number agreement are—for many European languages—characteristic examples of grammatical processes where morphosyntactic elements of one part of a phrase or sentence systematically covary with other elements (Bybee, 1985). Electrophysiology is well-suited to study how these relations are recovered, especially in language learners, because evoked responses measured with either EEG and MEG have sufficient temporal resolution to distinguish brain responses to individual words as they occur within a sentence (Osterhout and Holcomb, 1992; Hagoort et al., 1993 and for a review see Kutas et al., 2006)—in contrast to slower hemodynamic measures such as fMRI or Near-InfraRed Spectroscopy (NIRS). In studies of grammatical learning, the response to a so-called “critical word” or morpheme within a sentence—the point in the phrase where the relation can be first recognized—can be measured before and after learning to better understand how changes in behavioral recognition are related to changes in the brain responses (Osterhout et al., 2006). A recent review by Caffarra et al. (2015), summarized some of the main Event Related Potential (ERP) components found in recent longitudinal or learning studies: To date, the electrical P600 in response to morphosyntactic violations have been seen in a variety of short-term learning settings, L1–L2 pairings, and experimental designs (Weber-Fox and Neville, 1996; Hahne, 2001; Rossi et al., 2006; Frenck-Mestre et al., 2008; Kotz et al., 2008; Sabourin and Stowe, 2008; Weber and Lavric, 2008; Dowens et al., 2010; Moreno et al., 2010; Dowens et al., 2011; Foucart and Frenck-Mestre, 2011; Pakulak and Neville, 2011; Schmidt-Kassow et al., 2011a,b; Zawiszewski et al., 2011; Foucart and Frenck-Mestre, 2012; Xue et al., 2013; Bañón et al., 2014; Lemhöfer et al., 2014; Tanner et al., 2014). Nevertheless, it is not the only component that reflects grammar learning.

The N400 component has also been used to characterize L2 learners (Weber-Fox and Neville, 1996; Proverbio et al., 2002; Kotz et al., 2008; Weber and Lavric, 2008; Guo et al., 2009; Tanner et al., 2009; McLaughlin et al., 2010; Zawiszewski et al., 2011; Foucart and Frenck-Mestre, 2012; Xue et al., 2013; Tanner et al., 2014). While native speakers show an N400 component in response to a semantic violation and a P600 component in response to a (morpho)syntactic violation, in some cases it has been shown that L2 learners (at very early stage of learning) show a N400 component in response to a syntactic violation (McLaughlin et al., 2010). This was interpreted to mean that in the first stages of L2 acquisition, learners rely on lexical-semantic processing strategies. Later studies (Tanner et al., 2009; McLaughlin et al., 2010), examined learners of German at the end of the first year of a formal course, and at learners at the end of the third year of a formal course. While the group of learners from the third year showed a P600 in response to morphosyntactic violations, the first year group showed a biphasic N400-P600 response. A further analysis showed that this biphasic N400-P600 response is not representative of all learners, but an artifact of averaging brain responses of all participants: One subgroup of participants showed an N400 while the other subgroup a P600. Moreover, the amplitude of P600 positively correlated with the d' scores of the judgment task. McLaughlin et al. (2010) reviewed more studies with this pattern and suggested that participants progress from a N400 to a P600 response, and that the response of each participant depends on the stage of grammatical learning.

Short-term changes to violation-evoked components, in the time span of weeks or months, have also been seen during grammar learning using sensor-level EEG. Similar to the present design, Davidson and Indefrey (2009a) studied how ERP components such as the P600 are related with grammar acquisition process in adults using text materials.

The results showed a P600-like effect during and following training for certain types of agreement violations in Dutch learners of German, similar to native German speakers. In a similar study, Davidson and Indefrey (2011) found P600 responses for both declension and gender violations using text materials. Davidson and Indefrey (2009b) studied the MEG response to phrase-order violations in German learners of Dutch. However, Davidson and Indefrey (2009b) examined the response to text-stimuli rather than auditory materials, and the grammar training was not carried out in the laboratory, but rather in a classroom in a longitudinal design, so it is not clear whether the outcome seen in that study will also hold for the more focused laboratory based training regime such as in Davidson and Indefrey (2009a, 2011).

In the present study we employed MEG with a focused learning task to investigate this issue. Other learning studies have examined sensor-level violation effects with spoken materials. Unlike serially-presented text materials, spoken materials must be segmented during comprehension, and in principle this could modulate the timing of grammatical acquisition because learners may have difficulty recognizing individual lexical items. Mueller et al. (2005) showed that training can lead to the emergence of ERP grammatical violation patterns similar to native speakers with auditory materials, although with certain differences. She trained German participants in a mini-version of Japanese and examined three types of grammatical violation (word category, case, and classifier viola- tion). Participants completed a pretest, a training period using both comprehension and production, and a final post-test. Trained participants reached high level proficiency over a period of 4–10 h in all the conditions, and native-like proficiency in two out of the three conditions (classifier and word category). For these two conditions a native-like P600 effect was observed after but not before training, but non-native speakers did not show an N400 effect that was present in the native speakers' response. A follow up study by Mueller et al. (2007) similarly found no differences in elicited P600 between native-speakers and trained learners. However, in canonical sentences an anterior negativity was found. Finally, Mueller et al. (2008) found that in a group of participants trained with pseudoword materials showed a native-like N400-P600 response. She argued that removing semantic load could free up resources for the processing of syntactic violations. In the present study, we presented Spanish learners spoken phrases that used nouns and adjectives that were Basque-Spanish cognates in order to reduce the lexical-semantic or phonological learning load and enable participants to segment the speech during learning. Notably, Spanish and Basque share most of their phonological segmental inventories, despite substantial differences in their grammatical systems.

Relatively few training studies have examined how connected speech is produced in a second language during learning. This is potentially an important omission in the literature because ordinarily language learners acquire grammatical proficiency via practice of both comprehension and production (e.g., as in Mueller et al., 2005). Using MEG, Hultén et al. (2014) trained Finnish participants in a miniature language fragment of an artificial language. For the training phase (4 days) participants saw a picture, and listened and read a corresponding sentence, and finally had to repeat it. The task was arranged so that participants had to produce the correct agreement inflection on the last word of the sentences. They performed the experiment both in the miniature language (L2) and in the native Finnish (L1). The results showed that neural networks involved in production shared resources for L1 and L2. However, based on increased amplitude response of left parietal cortex and angular gyrus in the novel language, Hultén et al. (2014) proposed that L2 speech production increased cognitive effort as compared to L1 language processing (c.f., Hanulová et al., 2011,). To better mimic the learning task usually involved in grammar learning, in the task we used in the present experiment, learners discriminated between grammatically-correct and -incorrect phrases, as well as learned to produce the correct form of the phrases in a picture-description task. Also, instead of comparing L2 learners to a separate group of native speakers (as in Mueller's studies), following Hultén et al.'s approach, we measured the response in both L1 and L2 of the same participants to better characterize the difference in response to the two languages.

Our main hypothesis is that when using cognate vocabulary grammatical rules can be incorporated rapidly. Neurophysiologically, we hypothesize that the brain networks that form the basis for L1 proficiency are engaged during the explicit learning of a new L2. The main prediction based on this is that once the learned rule has been incorporated to real-time language processing, left posterior areas - which show a greater amplitude evoked response to a grammatical violation in the L1–will also be the areas that exhibit a greater amplitude violation response in the L2. Nevertheless, as initial stages of L2 learning remain unstudied with MEG we are not in a position to strongly assume the areas involved and restrict our analysis to those. This is the reason why we present here an exploratory study: We do not constrain our source-level statistical analyses to any region of interest. We are aware that this decision diminishes the statistical power; nonetheless, we want to observe and describe patterns in the data, even if they are not strongly supported by statistic. These patterns may then serve as priors that would help to develop confirmatory studies in this relatively understudied field.

## Materials and methods

### Ethics statement

The study was carried out at the Basque Center for Cognition, Brain and Language, and it was approved by its institutional review board (Ana Fernandez, Elena Salillas, Pedro Paz-Alonso, Monika Molnar).

### Participants

Seventeen (9 female, 8 male) healthy native speakers of Spanish aged 20–29 participated in the current experiment. Participants were right-handed Spanish native speakers with no (substantial) knowledge of Basque and reported no hearing or reading disorders. The participants were recruited from Donostia-San Sebastian or the surrounding communities. All participants were screened for magnetic interference prior to data collection, and provided informed consent (Declaration of Helsinki) before starting the experiment. Additional data from two subjects was recorded, but the data were discarded from the analysis because the participants did not follow the instructions.

### Mini-basque

**Figure 1:**
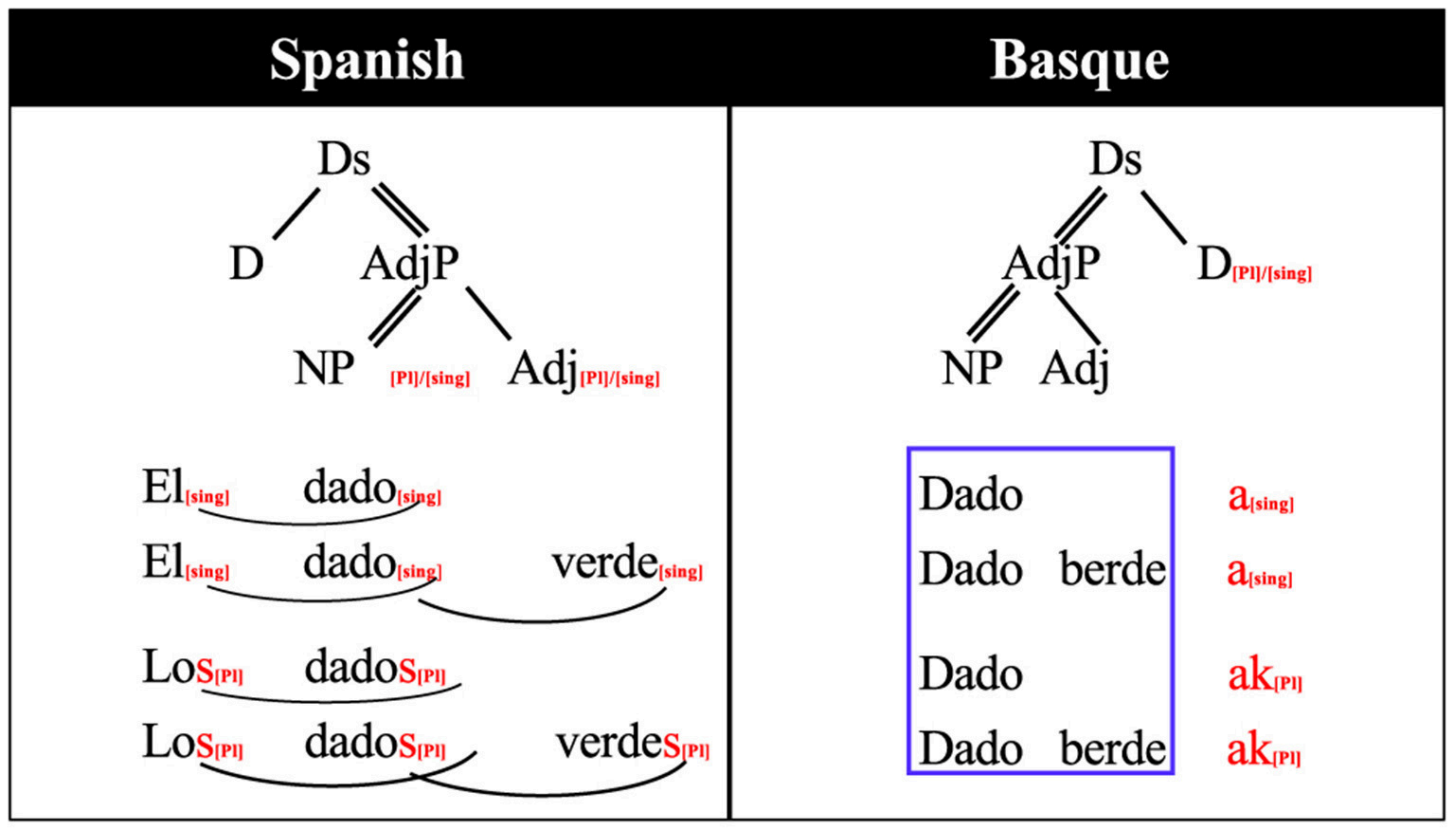
**Grammatical rule differences between Spanish and Basque number inflection for noun phrases**. dado: die, verde/berde: green, el/los/a/ak: the, D: determiner, DS: determiner structure, Adj: adjective, AdjP: adjective phrase, NP: noun phrase, Sng: singular, Pl: plural. Arrows indicate marking relationship, thin lines dependency, double lines head dependency. See Table 1 for a translation.

For this study we used a small fragment of Basque, which we will term “mini- Basque.” We exploited the fact that Spanish and Basque share a relatively large proportion of their vocabularies, but they differ substantially with respect to grammar. For the stimuli, we constructed Basque noun phrases that would contain new grammar rules for the participants but did not require learning new words, as the words were chosen to be already familiar to the subjects from their Spanish. In total, we chose 80 nouns and four adjectives that are phonologically similar to Spanish^1^. Also, we selected a grammatical relation (number marking) that would be relatively simple for participants to learn in the span of a few experimental sessions. Grammatical number is marked in both languages, but is implemented in a different way in Basque due to the different head directionality and the phrase ordering. In terms of the classification used by Caffarra et al. (2015), number inflection is implemented “differently.” As it is described in Figure 1, Spanish constructs plural determiner phrases marking the number in all the elements of the phrase. On the other hand, Basque only marks number on the last element of the phrase.

### Stimuli

We used three types of noun-phrases: Control phrases, violation phrases, and fillers (See Table 1).

**Table 1:** **Example phrases**.

**Condition**	**Mini-Basque Phrases**	**Spanish Phrases**
Control phrases	Dado[] | berdea [Sing]	El[Sing] dado [Sing] verde | [Sing]
	*The green die*	*The green die*
	Dado[] | berdeak[Pl]	Los[Pl] dados[Pl] verde | s[Pl]
	*The green dice*	*The green dice*
Violation phrases	^*^Dadoa[Sing] | berdea[Sing]	^*^El[Sing] dado[Sing] verde | s[Pl]
	^*^The green die	^*^The green die
	^*^Dadoak[Pl] | berdeak[Pl]	^*^Los[Pl] dados[Pl] verde | [Sing]
	^*^The green dice	^*^The green dice
Fillers	Dadoa[Sing]	El[Sing] dado[Sing]
	*The die*	*The die*
	Dadoak[Pl]	Los[Pl] dados[Pl]
	*The dice*	*The dice*

*| Denotes the violation point and the corresponding control point. Feature marking is shown in brackets (e.g., [Pl] or [Sing]), with underspecification in empty brackets: []*.

As it can be seen from both Figure 1 and Table 1, in Spanish, number agreement is marked by inflecting all the elements of the noun phrase: The determiner, the noun and the adjective: Los dados verdes (the green dice). The Spanish violation phrases were created by marking an incorrect number inflection of the adjective, therefore, the violation/control critical points come near to the end of the adjective. In contrast, Basque number is indicated only at the last word of the phrase (in this case, it could be the adjective or the noun): Dado berdeak (the green dice). The critical morpheme for the violation phrases is the first morpheme of the following adjective because it indicates the adjective follows an inflected noun.

We also added a filler condition consisting of simple noun phrases in Basque so that participants could not simply detect violations only on the basis of an inflected noun. In this case the noun phrases were composed of a noun, determiner, and number inflection, but no adjective. This obliges the participants to wait and listen if any adjective follows the marked-noun before deciding if it is a correct or incorrect phrase. In Basque, the determiner is not optional, so no trials consisting of a bare noun (e.g., dado) were presented. Trials from the filler condition were not included in the electrophysiological analysis.

Each of the comprehension test/training blocks consisted of 184 trials: 80 control phrases, 80 violation phrases, and 24 fillers. Violation, control and filler phrases were randomized inside each block, but the same list was presented for all participants. Each block lasted approximately 15 min.

The phrases of mini-Basque consisted of 80 nouns and four adjectives chosen to be phonetically similar to Spanish words. Sixty of these nouns and two adjectives were used for the training blocks and the rest of the 20 nouns and two adjectives for the generalization blocks. Thus, the same phrase was never heard twice, and the words that were used in training blocks were not present in the generalization tests.

Stimuli were recorded in a sound-proof booth using a female voice. Resulting audio files amplitude was equalized to the same loudness and presented to participants at 65 DB. The length of the recorded stimuli per block and condition can be found at Table 2.

**Table 2:** **Mean (std) of stimuli length (in seconds) per block and condition**.

**Block**	**Control**	**Violation**
Pre-test BQ	1.32 (0.23)	1.48 (0.29)
Pre-test SQ	1.56 (0.29)	1.61 (0.26)
Training 1	1.42 (0.32)	1.54 (034)
Training 2	1.43 (0.29)	1.60 (0.38)
Traning 3	1.41 (0.28)	1.58 (0.36)
Gen-test BQ	1.43 (0.25)	1.58 (0.31)
Post-test SP	1.61 (0.29)	1.65 (0.35)

Because the stimuli were presented auditorily the time point for each trial's critical morpheme varied. In order to establish the critical point, we calculated the average critical point for Spanish and Basque separately. For Basque we measured the onset of the second word using the software Praat (Boersma and Weenik, 1990, version 5326), and then the values were averaged. For the Spanish, the offset of the last word was measured (also using Praat), and then the values were averaged.

During MEG recordings the auditory stimuli were presented via panel speakers (two SSHP 60 × 60 panels; Panphonics Oy, Helsinki, Finland).

### Design and tasks

The study was carried out in two experimental sessions conducted on 2 consecutive days. There were two main reasons to go for a 2-day design. First, to test whether any effects seen on the first day remain until the second session. Second, in order to study the evolution of brain responses during training, a substantial number of trials are needed. If all were presented in the same session, the experiment would become too long and tiresome for the participants.

**Figure 2:**
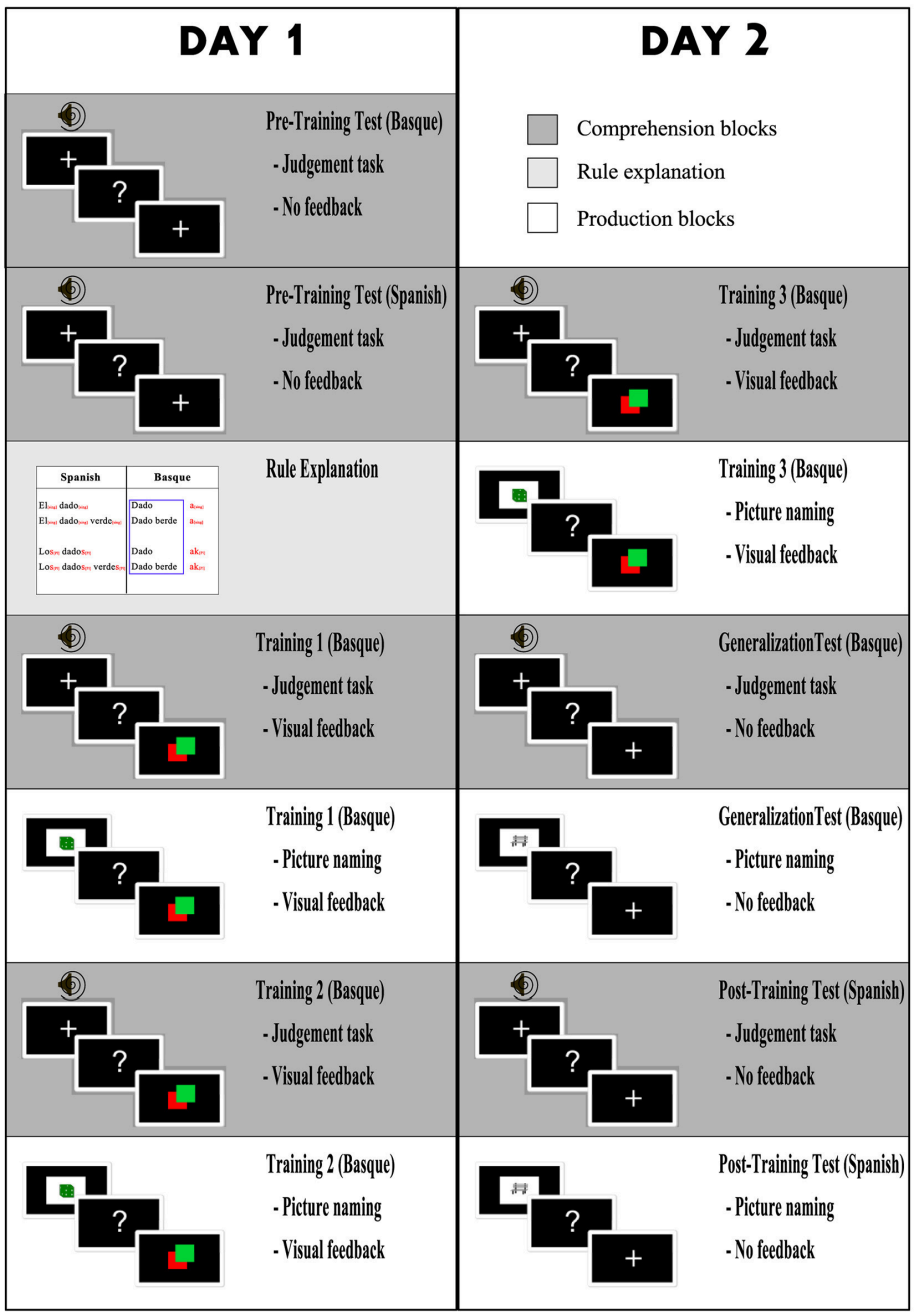
**Design of the study**. Left column contains the tasks of the first session, right column tasks of the second session (Session 1 and 2 were conducted on consecutive days). Dark gray blocks are comprehension blocks (grammaticality judgement task). White blocks are production blocks (picture naming task) and light gray block is grammar rule explanation.

The first session included seven blocks: A pretest in Basque, a pretest in Spanish, rule explanation, training on comprehension, training on production, training on comprehension again, and training on production again (see Figure 2, column 1).

The comprehension tasks in the experiment consisted of a series of trials in which a fixation cross appeared for 2 s on a back-projection screen, followed by the presentation of the auditory sentence via the panel speakers. The fixation cross remained on screen during the playback, and following the termination of a phrase, a question prompt appeared on the screen, and remained onscreen until the participant's response or a time out (4 s). During training blocks, the response was followed by a feedback display consisting of a green (correct) or red (incorrect) square. Feedback was provided based on the first response, the rest of the responses (if any) were discarded.

The pretest in Basque was conducted to confirm that participants' knowledge of Basque was minimal, and to measure whether there were any physiological effects due to stimuli differences between conditions. In the pretest, participants were told that they would hear Basque phrases and that they were to indicate if the phrases they heard were correct Basque or not by pressing a green or red button, respectively. The Spanish pretest was conducted in order to measure a baseline response in the participants' L1 that would allow later comparisons with Basque blocks. In this part, participants were instructed to indicate if the phrase they heard (in Spanish) was correct or incorrect, again by pressing a green or red button. Participants did not receive feedback in any of the pretest blocks. After both pretests were completed, participants were told that the remainder of the study consisted of a rule-learning task in Basque. In order to explain the rule the example of Figure 1 was used. Participants were allowed to ask as many questions as they wanted to ensure they understood the rule. Following the rule explanation and an explanation of the task, the training phase in the MEG started. For the comprehension blocks, participants listened to the Basque phrases and had to decide if the phrase was correct or incorrect, just as in the pretest. However, unlike the pretest, on each trial participants got visual feedback in order to allow self-correction.

The production task consisted of a picture description task. Participants first saw a fixation cross for 2 s, followed by an image with either one or two drawings presented in one color (e.g., a single green die, or a pair of green dice) on the back- projection screen for 3 s. After the image, a question mark prompt appeared for 7 s, and participants produced either a singular or a plural noun phrase. They received visual feedback in order to allow self-correction, and then the following trial began. Feedback was provided based on the first response, the rest of the responses (if any) were discarded. The production tasks on the training blocks were done in Basque.

The second session included six components: (1) training in comprehension, (2) training in production, (3) generalization test in comprehension, (4) generalization test in production, (5) comprehension test in Spanish, and (5) a production test in Spanish (see Figure 2, column 2). The training blocks were presented in the same sequence as the previous day. For both the generalization comprehension and generalization production tests in Basque, the same structure and instructions were used as in the training blocks, but no feedback was provided. Moreover, the stimuli used (explained in the previous section) were different from the stimuli used in training. The aim of the generalization blocks was to assess whether participants had learned to apply the grammar rule to new words rather than memorizing that particular noun-phrases were correct or incorrect. The Spanish comprehension and production blocks were completed to have a baseline in L1 for both production and comprehension for further comparison with Basque.

### Procedure

The two sessions were recorded on 2 consecutive days. All the blocks described in the design section were recorded for each participant. During the recordings, participants were asked to relax and acquire a comfortable position between blocks in order to prevent movements during data acquisition; they were instructed to avoid head, body and eye movements during the task. Two (vertical and horizontal) EOG channels were recorded for later artifact rejection, and a single bipolar ECG lead was recorded for heartbeat removal by ICA.

A Polhemus Isotrak (Polhemus, Colchester, VM, USA) was used to digitalize the head shape (around 120 points for each subject) and the fiducials in order to be able to align the head to each subject's structural MRI (T1 image). Additionally, five head localization coils were attached to the participant's head, and their spatial location (relative to fiducials) was recorded. The five coils were active during the MEG recording to provide continuous head position information (cHPI). The MEG data was acquired with a 1000 Hz sampling rate and filtered during recording with a high-pass cutoff at 0.03 Hz and a low-pass cutoff at 330 Hz via the Elekta acquisition software.

Audio files were presented (65 dB at subject head position) via panel speakers (two SSHP 60 × 60 panels; Panphonics Oy, Helsinki, Finland.) and any visual presentation was done via a back-projection screen.

### Data analysis

#### Behavioral analysis

The behavioral response data corresponding to comprehension blocks were modeled using a multilevel generalized linear regression model (Dixon, 2008) using the factors condition (violation vs. control) and block (e.g., pre-test, training 1,…). The coefficients from the multilevel analysis were back-transformed to proportions.

The data from the production blocks were similarly modeled using a multilevel generalized linear regression model (Dixon, 2008) using the factor block (e.g., pre-test, training 1,…). The aim of this study is to examine the grammar acquisition and not the lexical acquisition. Therefore, a response was considered correct when the grammar rule was correctly applied even if the intonation, pronunciation of the noun or the adjective was not completely correct. For example, if participants used the Spanish form of the cognate but the Basque grammatical rule, the response was considered correct, with respect to grammar.

#### MEG data

The design contained both comprehension and production tasks. In order to show that participants acquired the rule correctly behavioral analysis reports results from both type of tasks. Nevertheless, the interest of the study is mainly on the discrimination of grammatical correctness of the sentences. Therefore, for the MEG data only the comprehension tasks were analyzed.

Using MaxFilter 2.2, the recorded MEG data were filtered using temporal Source Space Separation (tSSS) with a 4 s time window and a minimum correlation of 0.98. Individual's head origins and bad channels were supplied manually, data were downsampled to 250 Hz, and line frequency (50 Hz) and its harmonics were filtered. Following recommendations from the MEG laboratory at MRC Cognition and Brain Sciences Unit (http://imaging.mrc-cbu.cam.ac.uk/meg/Maxfilter_V2.2), the downsampling and the filtering were conducted in two separated steps. Head origin of each participants was transformed to default position, to ensure that head position were standardized across participants and blocks, on average heads were shifted 20.4 mm (std = 6.65) on the first day and 22.8 mm (std = 5.01) on the second day. The data were processed using Fieldtrip toolbox (version 20141202, Oostenveld et al., 2011), all the analysis was conducted only using the gradiometer sensors (the magnetometers were discarded due to noise reasons). First, data were segmented into epochs. The onset of the epoch was locked to the onset of the trial for both Basque and Spanish. Data were segmented into 4 s epochs consisting of 1 s before the onset of the trial and 3 s following the onset of the trial.

Then, the data were screened for jump artifacts, epochs with a z-value larger than 20 were automatically rejected. Each epoch was padded to 12 s and then filtered with a low-pass FIR filter at 40 Hz (one pass zero-phase), and the resulting epochs were baselined corrected with respect to a 200 ms interval (–200 to 0 ms from trial onset).

The data were decomposed using the fast Independent Component Analysis (fast ICA) algorithm, the number of components that were calculated equal the number of gradiometers (204) and prior to the analysis no data dimension reduction algorithm was applied. The fastICA algorithm was applied to uncombined gradiometers (204 sensors). Then, the correlation of each ICA component time-course with the HEOG, VEOG, and ECG time-course was calculated. The components whose correlation exceeded three standard deviations of the mean correlation in any of the cases (HEOG, VEOG, or ECG) were zeroed out before back projecting the single-trial data into the original sensor space. The uncombined gradiometers were then averaged according to experimental condition (violation vs. control). Once averages were calculated, the planar gradiometers were combined. Only trials associated with correct behavioral responses were included in the violation control ERF contrasts, except for the Basque pre-test block where all clean trials were included (average number of included trials per block are provided in Table 3). The differences between conditions were assessed using a clustering and randomization test (Maris and Oostenveld, 2007). A randomization distribution of cluster statistics was constructed for each subject over time and sensors and used to evaluate whether there were statistically significant differences between conditions over participants for each violation-control comparison in a phase of training (e.g., pre-test, training, post-test, etc.). In particular, t-statistics were computed for each sensor and time point during the [–0.2, 2.5] time window, and a clustering algorithm formed groups of channels over time points based on these tests. The neighborhood definition was based on the template for combined gradiometers of the Neuromag-306 provided by the toolbox. In order to a data point to become part of a cluster, they were thresholded at *p* = 0.05 (based on a two-tailed dependent *t*-test, using probability correction) and it had to have at least two neighbors. The sum of the *t*-statistics in a sensor group was then used as a cluster-level statistic (e.g., the maxsum option in Fieldtrip), which is then tested using a randomization test using 1000 runs [the standard procedure is to use 500 runs, however when this results in clusters with *p*-values close to the threshold it is recommended to double the run number (1000 runs) to disentangle if the cluster is below or up the threshold].

**Table 3:** **Mean number (STD) of trials included in the MEG analysis of the comprehension tasks per block**.

**Block**	**Control**	**Violation**
Pre-test BQ	77.8 (2.0)	78.7 (1.8)
Pre-test SP	73.2 (7.0)	73.2 (7.4)
Training 1	72.8 (4.7)	70.5 (7.2)
Training 2	69.9 (18.5)	69.9 (18.7)
Training 3	76.4 (3.7)	72.7 (10.8)
Generalization BQ	74.8 (3.6)	72.4 (6.6)
Post-test SP	73.8 (3.6)	72.4 (6.6)

For simplicity, the visualization of the results shows the ERF waveforms of all sensors, and the topography plots show the raw average of the violation-control difference of the statistically most significant cluster. If a sensor was part of the cluster during any point of the window (not necessarily the whole time window), it appears highlighted on the topography plot.

Because the stimuli were presented auditorily the time point for each trial's critical morpheme varied. In order to establish the critical point, we calculated the average critical point for Spanish and Basque separately. For Basque we measured the onset of the second word using the software Praat ((Boersma and Weenik, 1990), version 5326), and then the values were averaged. For the Spanish, the offset of the last word was measured (also using Praat), and then the values were averaged.

For the source level data analysis, Minimum Norm Estimate (MNE) (Dale et al., 2000) was used. Structural MRI (T1 images) were segmented into scalp, skull brain and CSF, and a volume conduction model was constructed based on this segmentation using a single shell approximation (Nolte, 2003) by assigning conductivity to the brain. This volume conduction model and a 5124-point mesh grid based on the canonical cortical sheet (available in Fieldtrid) were used to construct the leadfields. These leadfields were pre-whitened before calculating the inverse solution. When the source covariance is estimated a scaling factor is applied (calculated automatically by Fieldtrip) in order to forth the source covariance to fulfill the next equation trace(A^*^R^*^A′)/trace(C)=1 where A is the leadfield, R the source covariance and C the noise covariance. The time courses for each mesh-vertex in the forward model was estimated using MNE with a regularization lambda value of three (for both lambda and lambda noise). After that, the three moments of the source time series were projected to their strongest orientation at each vertex.

Statistical analyses were also conducted on the source level data to identify which areas contributed to the differences seen at the sensor level. In source space, a clustering and randomization test (500 runs) was used similarly to the sensor level analysis (Maris and Oostenveld, 2007). In particular, t-statistics were computed for each vertex and on an averaged time window selected for each block based on sensor level-analysis, and a clustering algorithm formed groups of vertex based on these tests. The neighborhood definition was defined based on a distance measure (maximum neighbor distance of 10). In order to a data point become part of a cluster, they were thresholded at *p* = 0.05 (based on a two-tailed dependent *t*-test, using probability correction) and it had to have at least 2 neighbors. The sum of the *t*-statistics in a sensor group was then used as a cluster-level statistic (e.g., the “maxsum” option in Fieldtrip), which is then tested using a randomization test using 500 runs. In order to simplify the visualization of the source-space we only show the regions that were statistically significant or near to significant. For the blocks with no significant cluster we showed the cluster with lower *p*-value, in order to show the trend of the data in that block. However, we are aware that the interpretation of these no-significant clusters should be cautious. The cortical regions' labels were defined based on the AAL atlas provided in Fieldtrip toolbox: First the AAL atlas defined in the MNI space was interpolated to the common source mesh. Then for each significant cluster we created a mask, and got the labels of the masked vertex from the interpolated atlas.

We also conducted an statistical analysis at source space without restricting it to a given time window. While the previous analysis would help us identifying the sources responsible of the effects picked at sensor level, we also want to explore any violation-control effect at source level that may have not been picked at sensor level.

For this unrestricted analysis, a clustering and randomization test (500 runs) was used similarly to the sensor level analysis (Maris and Oostenveld, 2007). In particular, t-statistics were computed for each vertex and time point during the [–0.2, 2.5 s] time window for each block and a clustering algorithm formed groups of vertex and time points based on these tests. The neighborhood definition was defined based on a distance measure (maximum neighbor distance of 10). In order to a data point become part of a cluster, they were thresholded at *p* = 0.05 (based on a two-tailed dependent *t*-test, using probability correction) and it had to have at least 10 neighbors. The sum of the *t*-statistics in a sensor group was then used as a cluster-level statistic (e.g., the “maxsum” option in Fieldtrip), which is then tested using a randomization test using 500 runs. In order to simplify the visualization of the source-space we only show the regions that were statistically significant or near to significant. Although the results section will report all these clusters (in order not to hide any information), based on Guthrie and Buchwald (1991) the discussion section will not take into account clusters which last less than 10 consecutive data points (i.e., 40 ms). Although this constraint it's still is not well-known in the MEG literature it is widely used in the EEG literature (Murray et al., 2001; Molholm et al., 2002; Kecskés-Kovác et al., 2013; Berger et al., 2014). For the blocks with no significant cluster we showed the cluster with lower *p*-value, in order to show the trend of the data in that block. However, we are aware that the interpretation of these no-significant clusters should be cautious. The cortical regions' labels were defined based on the AAL atlas provided in Fieldtrip toolbox: First the AAL atlas defined in the MNI space was interpolated to the common source mesh. Then for each significant cluster we created a mask, and got the labels of the masked vertex from the interpolated atlas.

## Results

### Comprehension training and test: behavioral analysis

Table 4 shows the proportion (95% CI) of ‘acceptable’ labels for the control and violation phrases of the comprehension task as a function of test phase. In all conditions except the Basque pre-test, participants had good discrimination between the violation and control phrases. They could correctly label the grammatical stimuli as acceptable with a proportion greater than 0.95 and they could exclude the errors in most cases. In the pre-test for Basque, however, participants labeled the control stimuli acceptable less than half the time (0.28), and correspondingly mislabeled the violation stimuli more than half the time (0.67). As a consequence, participants' accuracy (i.e., the proportion correct in the violation and the control conditions) was lower than chance in the Basque pre-test (this response bias is discussed in section Pre-test Basque), but near ceiling in the other blocks.

**Table 4:** **Proportion (95% CI) of' ‘acceptable’ labels for the control and violation phrases of the comprehension task as a function of test phase**.

	**Control (CI)**	**Violation (CI)**
**PRE-TEST**		
Spanish	0.95 (0.94, 0.96)	0.04 (0.02, 0.07)
Basque	0.28 (0.21, 0.35)	0.67 (0.55, 0.77)
**TRAINING**		
1	0.95 (0.93, 0.97)	0.09 (0.05, 0.16)
2	0.97 (0.95 0.98)	0.05 (0.02, 0.12)
3	0.99 (0.98, 1.00)	0.03 (0.01, 0.1)
**GENERALIZATION**		
Basque	0.99 (0.98, 0.99)	0.03 (0.01, 0.09)
**POST-TEST**		
Spanish	0.98 (0.97, 0.99)	0.05 (0.01, 0.14)

*Proportions are taken from a mixed-effects model of the single-trial behavioral data*.

### Production training and test: behavioral analysis

Table 5 shows the proportion of (95% CI) correct phrases produced for the singular and plural conditions as a function of test phase. In all conditions, participants produced phrases that we labeled as grammatically correct or incorrect, we judged the correctness only based on the use of the new acquired rule and no other aspects of the production (such as pronunciation or the preciseness of the vocabulary). They could correctly produce the grammatical phrases for both singular and plural conditions at a level greater than 0.96 (see Table 5).

**Table 5:** **Proportion of (95% CI) correctly produced phrases for the singular and plural conditions as a function of test phase**.

	**Singular (CI)**	**Plural (CI)**
**TRAINING**		
1	0.96 (0.94, 0.98)	0.97 (0.91, 0.99)
2	0.98 (0.97, 0.99)	0.98 (0.94, 1.00)
3	0.99 (0.98, 1.00)	0.99 (0.93, 1.00)
**GENERALIZATION**		
Basque	0.99 (0.98, 1.00)	0.99 (0.89, 0.99)
**POST-TEST**		
Spanish	1.00 (0.99, 1.00)	0.99 (0.92, 1.00)

*Proportions are taken from a mixed-effects model of the single-trial behavioral data*.

### Comprehension training and test: electrophysiological analysis

#### MEG-sensor level

**Figure 3:**
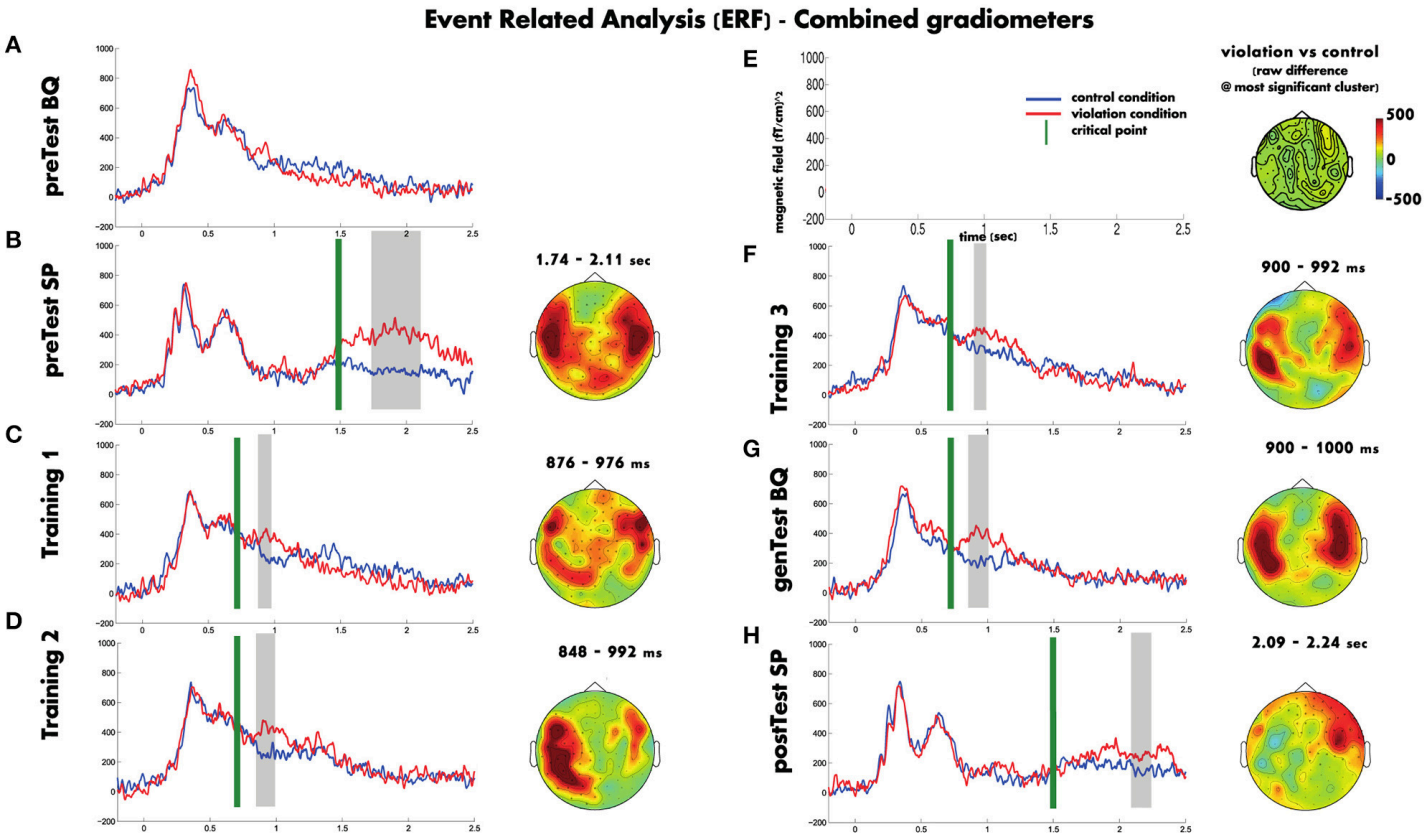
**The plotted waveforms represent the averaged ERFs across all the sensors, the gray boxes the time windows where the cluster was significant and the topography plots show the raw difference between the violation and control conditions in those time windows**. In order to simplify the visualization Figure 5 shows only the cluster with lowest *p*-value for each block. Blue waveforms correspond to the control condition, red waveforms to the violation condition. Green vertical line denote the average critical point of each block. Gray boxes indicate statistically significant time windows. The topography plots show the raw difference between the violation and control for the cluster with the most supporting evidence of that block. Blocks appear on the order they were run **(A–H)**, with the scales given in **(E)**.

Figure 3 shows a summary of the ERF analysis (average number of included trials per block are provided in Table 3). In the Spanish pretest (Figure 3B), the onset of the sentence does not differ between violation and control conditions. Only after the critical point both conditions do start diverging, where the violation phrases had a greater amplitude response than the control phrases and the difference lasted ~1 s. However, the statistical analysis supports the difference in a smaller time window. The analysis revealed a cluster only between 240 and 600 ms after the averaged critical point, that includes bilateral temporal and posterior sensors (clusterstat = 6686; *p* < 0.002).

Figure 3A shows that during the Basque pretest there were similar responses for both the violation and control phrases. The statistical analysis did not show any significant cluster (clusterstat < 250; *p* > 0.2).

Regarding the training blocks, in all three the violation and control conditions do not differ before the average critical point occurs. After this point the violation phrases had a greater amplitude response than the control phrases and the difference lasted approximately 100 ms. Although in the time domain the three training blocks show similar patterns, in the spatial domain there is an evolution from block to block. In the first training block (see Figure 3C) the effect was spread through almost all the sensors but the largest difference was found on a group of right frontal sensors and also on some left posterior-temporal sensors between 29 and 126 ms after the mean critical point (clusterstat = 966.5; *p* = 0.004). On the second training block (see Figure 3D), the effect was less widespread and it mainly appeared on the left hemisphere between 0 and 142 ms after the mean critical point (clusterstat = 1422; *p* = 0.002). And finally, in the third training block (last block with feedback, see Figure 3F), the effect was localized on both left and right temporal sensors between 50 and 142 ms after the mean critical point (clusterstat = 375.5; *p* = 0.048).

The Basque generalization test (see Figure 3G) showed a response similar to the one found in the third training block, the magnitude of the effect was maintained and the topography involved sensors of both hemisphere temporal areas. The statistical analysis revealed a significant cluster between 50 and 150 ms after the mean critical point (clusterstat = 1220; *p* = 0.008) formed by right temporal sensors.

Finally, the Spanish post-test (see Figure 3H) showed a different pattern from the Spanish pre-test. Both conditions started to diverge later than in the pre-test block and the magnitude of the difference was smaller than in the Spanish pretest. Moreover, although the topography was quite widespread, the main difference was located on right frontal sensors. The statistical analysis showed a significant cluster (clusterstat = 1514; *p* < 0.002) consistent with the effect we described, between 586 and 736 ms after the mean critical point, formed mainly by frontal sensors from the right hemisphere.

#### MEG-source level

##### Time-constrained statistical analysis

**Figure 4:**
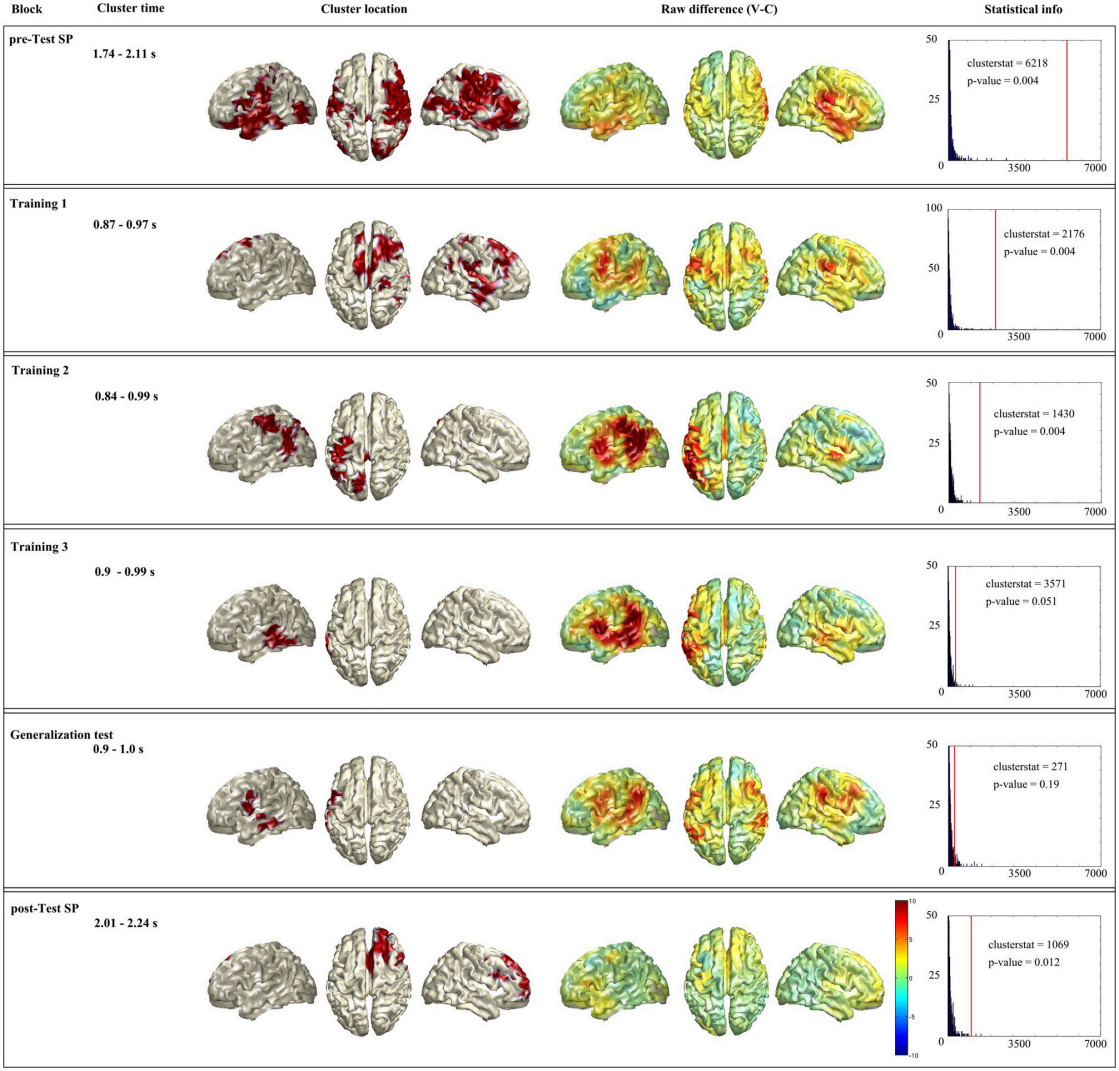
**Summary of the time-restricted analysis**. Each horizontal box shows the significant cluster of a block (when a block had no significant cluster the one with lowest *p*-value is presented). First column shows the name of the block, second column the time window used for the analysis. Third column shows the source localization of the cluster. Fourth column shows whole brain raw difference between conditions (violation-control) for the given time window. And last column shows the statistical information of the given cluster: In blue the histogram of the random distribution, and the red line denotes the cluster stat value of that cluster.

Figure 4 summarizes the time-constrained statistical analysis. First column shows the name of the block, second column the time window used for the analysis (remember that this time window was based on sensor level analysis). Third column shows the source localization of the cluster. Fourth column shows whole brain raw difference between conditions (violation-control) for the given time window. And last column shows the statistical information of the given cluster. As it has been explained in the Methods section, remind that the labels of the regions come from the AAL atlas.

In the Spanish pre-test (see Figure 4, first row), the statistical analysis showed a significant difference between violation and control responses coming from a cluster (clusterstat = 6218; *p* = 0.004) formed by spread bilateral network. The network included right superior motor area, right frontal superior are and both left and right: Parietal lobe (superior parietal, pre- and post-central gyri), frontal lobe (orbitalis, opercularis, and triangularis), temporal lobe (Heschl's, middle and superior temporal gyri and middle and superior temporal pole), parieto-occipital areas (supramarginal and angular gyrus), fusiform, and middle occipital lobe. This cluster is consistent with the spread topography found at sensor level.

For the first training block in Basque (see Figure 4 second row), the statistical analysis showed a significant difference between violation and control responses coming from a cluster (clusterstat = 2176; *p* = 0.004) located mainly in the right hemisphere. The cluster includes right parietal lobe (superior motor area, pre- and post-central gyri), frontal lobe (middle and superior frontal gyri, triangularis opercularis), parieto-occipital areas (supramarginal and angular gyrus), temporal lobe (Heschl, inferior, middle and superior temporal gyri and middle, and superior temporal pole), fusiform and left superior motor area. This pattern is also consistent with the sensor level topography which shows the effect mainly on right frontal and temporal sensors.

Regarding the second training block in Basque (see Figure 4 third row), the statistical analysis showed a significant difference between violation and control responses coming from a cluster (clusterstat = 1430; *p* = 0.004) located mainly in the left hemisphere. The cluster is formed by left parietal lobe (superior parietal area, pre- and post-central gyri), temporal lobe (inferior, middle and superior gyri), parieto-occipital areas (supramarginal and angular gyrus), superior occipital area and fusiform. This pattern is also highly consistent with the sensor level analysis.

In the third training block (see Figure 4 fourth row), the statistical analysis showed a near to significant difference between violation and control responses coming from a cluster (clusterstat = 357, *p* = 0.051) in the left hemisphere. This cluster is formed by left temporal lobe (inferior, middle, and superior gyri), inferior occipital gyri and fusiform. In this case there is a small mist-match with the sensor level. The sensor level topography shows a bilateral effect on both hemisphere temporal sensors, while the statistical analysis at sensor level picks only the right hemisphere sensors, the statistical analysis at source level picks up the left hemisphere regions.

The source level statistical analysis of the generalization test in Basque (see Figure 4 fifth row), does not show any significant cluster. Therefore, we show here the cluster that is closest to the significance (clusterstat = 271; *p* = 0.19) in order to at least show the trend of this block. This cluster is formed by left parietal lobe (opercularis, pre- and post-central gyri) and temporal lobe (inferior, middle, and superior gyri). This pattern is consistent with the sensor level topography, although we want to remind that is not a significant result, and that we should carefully interpret it.

Regarding Spanish post-test (see Figure 4 sixth row), the statistical analysis revealed a significant cluster (clusterstat = 1069; *p* = 0.012) located mainly in the right hemisphere. It is formed by right parietal lobe (pre-central gyrus and superior motor area) frontal lobe (opercularis, triangularis, orbitalis, middle, and superior-frontal gyri) and left superior motor area. This pattern is highly consistent with the sensor level analysis, although it differs from the pattern at Spanish pre-test.

##### Unconstrained statistical analysis

**Figure 5:**
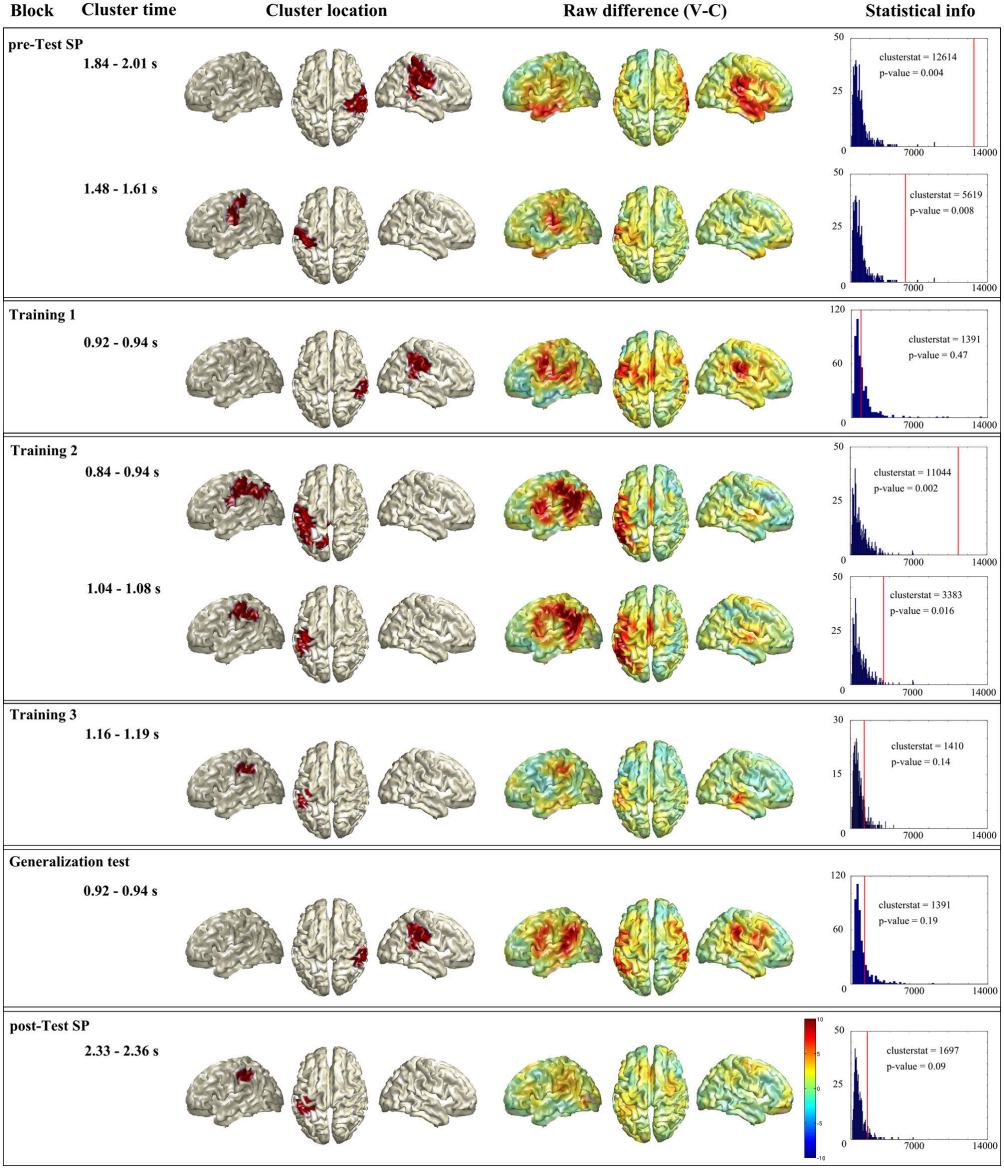
**Summary of the unrestricted analysis**. Each horizontal box shows the significant cluster(s) of a block (when a block had no significant cluster the one with lowest *p*-value is presented). First column shows the name of the block, second column the time window where the cluster was present. Third column shows the source localization of the cluster. Fourth column shows whole brain raw difference between conditions (violation-control) for the given time window. And last column shows the statistical information of the given cluster: In blue the histogram of the random distribution, and the red line denotes the cluster stat value of that cluster.

Figure 5 summarizes the unrestricted statistical analysis. First column shows the name of the block, second column the time window where the cluster was present. Third column shows the source localization of the cluster. Fourth column shows whole brain raw difference between conditions (violation-control) for the given time window. And last column shows the statistical information of the given cluster. As it has been explained in the Methods section, remind that the labels of the regions come from the AAL atlas.

In the Spanish pre-test (see Figure 5 first box), the statistical analysis showed two significant clusters. The first cluster (clusterstat = 12614; *p* = 0.004) appeared between 1.84 and 2.01 s and was localized at right parietal lobe (supramarginal, pre- and post-central gyri) and superior temporal gyrus. The second cluster (clusterstat = 5619; *p* = 0.008) appeared between 1.484 and 1.612 s and was localized at left parietal lobe (inferior parietal area and post-central gyrus).

For the first training block in Basque (see Figure 5 second box), the unrestricted analysis did not reveal any significant cluster. The cluster with smallest p-value (clusterstat = 1391; *p* = 0.47) appeared between 924 and 940 ms and was localized at right inferior parietal lobe supramarginal, pre- and post-central gyri and superior temporal gyrus).

Second training block (see Figure 5 third box) gave two significant clusters. The first one (clusterstat = 11044; *p* = 0.002) was found between 844 and 944 ms and was formed by left parietal lobe (supramarginal, pre- and post-central gyri) and left angular gyrus. The second cluster (clusterstat = 3383; *p* = 0.016) was found between 1.044 and 1.08 s and located at left inferior parietal area, supramarginal and angular gyri.

In the third training block (see Figure 5 fourth box) there was no significant cluster. The cluster with lowest *p*-value (clusterstat = 1410; *p* = 0.14) appeared between 1.16 and 1.19 s and was located at left parietal lobe (supramarginal, pre- and post-central gyri).

The generalization block (see Figure 5 fifth box) did not show a significant cluster neither. The cluster with lower *p*-value (clusterstat = 1391; *p* = 0.19) was found between 924 and 940 ms and located at right parietal lobe (inferior parietal area, supramarginal, and post-central gyri) and superior temporal gyrus.

Finally, the Spanish post-test (see Figure 5 sixth box) gave a marginally significant cluster (clusterstat = 1697; *p* = 0.09) between 2.33 and 2.36 s and located at left parietal lobe (inferior parietal area, supramarginal, and post-central gyri).

##### Summary of both source level analysis

In general we see that the unrestricted statistical analysis has less statistical power than the restricted analysis. Moreover, when we compare directly the clusters obtained in both analysis although some patterns could be considered similar, most of the clusters do not overlap on location. The reader could assume, that both analysis are telling completely different stories and that we should find a measure that lets us decide forward one or the other.. However, when we compared the clusters obtained in each type of analysis with the raw difference between violation and control conditions and/or the sensor level data, we see that all the clusters are consistent with a given part of the bigger pattern. Therefore, we see that each analysis is sensitive to different parts of the violation-control effect. The two analysis are not telling us a completely different story, but giving us a different part of the story, and therefore should be taken as complementary results that would help us understand better what is going on.

## Discussion

The present study investigated ERFs and their source localization related to morphosyntactic number agreement during the training sessions and a generalization test in adult learners of a simple grammar fragment. In general, the results showed that ERFs can change quickly during SLA, and that the violation-related responses related to the discrimination of correct and incorrect Basque phrases can emerge within hours of training. The sources of these violation-related responses appear to be localized in areas broadly similar to native language responses, when considered at a whole-brain scale of analysis. Below, we discuss the results from each block separately before the general discussion.

### Pre-test basque

Participants' accuracy on the pre-test block was below chance, suggesting that participants were either choosing randomly with some bias, or systematically misclassified the stimuli. While it is counterintuitive that accuracy was below chance, participants appeared to classify the stimuli according to the surface morphology rather than an underlying rule. Although this is speculative, it may be that in this type of task, Spanish speakers are more likely to classify a phrase as grammatical Basque to the extent that the phrase includes Basque-specific inflectional morphology (e.g.,'-a' or'-ak') not found in Spanish. Nonetheless, the absence of differences in the ERF waveforms between the violation and control conditions suggests that there was no cue in the stimuli (e.g., prosodic or phonological differences) that would differentiate the conditions.

### Pre-test spanish

As expected, the accuracy in this block was at ceiling, confirming that participants were paying attention to the task and following the instructions. We found that the ERFs followed a similar time course and sensor-topography in both the violation and the control conditions, with a greater response magnitude in the violation condition. We found no statistical differences between the violation and control conditions until 240 ms after the critical point, and then the violation response was stronger compared to the control condition for around 1 s. The difference was mainly found on bilateral temporal sensors, although it was present also in some parietal and occipital sensors. Consistent with the sensor-level analysis, the source-level analysis showed that the effect was localized mainly at the bilateral parietal and temporal lobes.

### Training

Learners achieved high performance beginning in the first training block, and there were corresponding ERF differences between the violation and control trials. The onset of the effect relative to the critical point was a little earlier than the Spanish pre-test timing, and the duration of the effect was shorter (150 vs. 360 ms in Spanish) in all three training blocks. Also, the spatial localization of these effects differed from block to block.

Based on the behavioral results, it might be assumed that if from the first training block performance is high, learning has already occurred and that the training blocks were not reflecting learning. However, this assumes that having knowledge is the same as applying knowledge in real time as sentences or phrases unfold. However, it has been already proposed that learners need time before they transfer the rule-based knowledge into their real-time language processing system (McLaughlin et al., 2010).

In the first training block the effect was mainly located on right parietal sensors and on a few left temporal and posterior temporal sensors. Both source analyses showed that the effect is mainly located at right fronto-parietal lobes and temporal lobe. Regarding the second training block, the effect was mainly found on left hemisphere sensors and some of the right frontal sensors. Source analyses showed a pattern consistent with the sensor-level analysis: At source level we see that the violation condition elicited a stronger response in the left parietal temporal lobes, and a bit of the occipital lobe. As for the third training block, similar to the second training block, the effect was mainly located at bilateral temporal sensors. The topography of this block resembles the topography of the Spanish pre-test block. Both source level analyses trend to locate the effect in left temporal lobe, inferior-occipital gyri, and fusiform. However, these results were marginally significant and the interpretation should be cautious. In this case there is a small mist-match with the sensor level. The sensor level topography shows a bilateral effect on both hemisphere temporal sensors, while the statistical analysis at sensor level picks only the right hemisphere sensors, the statistical analysis at source level picks up the left hemisphere regions.

The first training block effect was localized in right frontal areas. This area has been previously related with non-syntactic specific error detection processes (Indefrey et al., 2001). Moreover, based on the HERA model (Habib et al., 2003), right prefrontal cortex is involved in memory retrieval while left prefrontal cortex is involved in memory encoding. The fact that right frontal areas seem to be the main sources of the effects found at this block, we suggest that on this first training block some memory retrieval process is involved in the judgment of grammaticality in early phases of grammar learning.

The second training block showed a left-lateralized effect although right frontal sensors show also some difference between the violation and control conditions. The third training block's sensor level topography showed that the effect is found at bilateral temporal areas, similar to the pattern we found in the Spanish pre-test. When moving to source-level analysis, similar to the second training block, the effect is mainly present in language-related areas but the pattern still differs from the Spanish pre-test (recall that the Spanish pre-test showed the effect on the both left and right hemispheres). From previous fMRI studies we know that the localized areas have been found to be part of the language network involved in speech comprehension (Friederici, 2011). Similarly, Davidson and Indefrey (2009b) studied the MEG response to phrase-order violations in German learners of Dutch, and a source reconstruction of that activity implicated a variety of left-hemisphere perisylvian areas showing a greater amplitude response to grammatical violations after months following formal coursework. The fact that the main effect occurs on language areas could suggest that the new L2 memories are being created and embedded in regions that already process lexical and grammatical information, rather than in separate regions that are responsible for other cognitive processes. However, even if these areas are language-related areas the effects found in these blocks still differ from effects found in Spanish (remember that Spanish pre-test showed the effect on both hemispheres) and their statistical support is only marginally significant. A possible explanation that could account for this effect could be that in these blocks participants are relying on a more automatized process than in the first training block but that it still differs from the one that is involved in their L1 and that this difference in proficiency is reflected in the amplitude of the evoked response.

Finally, one can probably not exclude intrinsic variability as an explanation for the block-to-block differences seen during training. While it was the case that the behavioral discrimination was at a high level for the participants, the magnitude of the violation effect was smaller and shorter in duration. Because smaller differences are associated with greater statistical variability, some of the block-to-block differences may be associated with the variability of the effect.

### Generalization test

The performance level showed on the three training blocks was maintained in the generalization test. The stimuli that were used in this test were formed by novel nouns and adjectives that had not been presented before during the training and the task did not provide any feedback to the participants. Therefore, it appears that participants were able to generalize the rule learned on the training blocks to novel phrases.

Regarding the neural correlates of this block, the ERF analysis showed an effect onset consistent with the previous blocks. The effect magnitude was comparable to second and third training block and Spanish pre-test, and the topography showed that the effect was mainly localized at bilateral temporal sensors similar to the third training block and the Spanish pre-test. However, the source level analyses did not give any significant result. The trend indicates that areas responsible of the effect could be some of the ares found in the Spanish pre-test: Left parietal lobe (opercularis, pre- and post-central gyri) and temporal lobe (inferior, middle, and superior gyri) and right inferior parietal area, supramarginal, and post-central gyri. However, we cannot throw a strong conclusion, due to lack of statistical power.

In this block two of the measures we have (behavioral and sensor level) show similar patterns compared to Spanish pre-test, suggesting that responses to morphosyntactic manipulations early in training can be shaped toward the L1, and generalized to novel words. Although it is not statistically significant, both source level analyses show a trend also toward L1-like pattern. Nonetheless, the timing of the effect in Basque occurs relatively early compared to Spanish effect. This kind of paradigm has not been much studied in Basque and, therefore, it is hard to compare the timing of the effect with previous literature. It may be the case that violation responses in Basque and Spanish do not coincide on time. We discarded that the effect in Basque could appear due to stimuli-differences because Basque pre-test shows no violation-control effect. Moreover, the timing of the effect is consistent across all the training blocks and the generalization test. This gives us confidence on believing that the effect found is a violation-control effect. However, we'll need to further study if the source-level absence of results is due to statistical power or because the effect is not really maintained in the generalization test.

### Post-test spanish

The behavioral results showed that participants performed well on the post-test obtaining similar scores compared to the Spanish pre-test, as expected.

Surprisingly, the ERF analysis showed differences between the Spanish pre-test and post-test. The post-test effect started considerably later (580 ms after critical points instead of 240 ms) and lasted only 150 ms (pre-test lasts around 360 ms). Moreover, the effect magnitude was reduced and the topography was different. The post-test effect was mainly localized on right frontal sensors and some left frontal sensors. The time-restricted source level analysis showed that right frontal areas are involved in the violation-control effect, however the non-restricted analysis showed a trend toward left parietal areas and a different timing.

There are different possible explanations for the discrepancy we find between Spanish pre- and post-test. On the one hand, the Spanish post-test was one of the last blocks of the session and participants could have been fatigued. Even if they gave the correct response, the route used for getting the response could be different. However, we discard this possibility, because as a reviewer suggested, if this was really a fatigue effect we should have notice it also in other blocks. Another possibility is that, the discrepancy could be a result of a switch effect. On the second session, participants went through a training block and the generalization test, what means that they were involved in the Basque tasks for around 1 h, and they then needed to perform the same task in their recently-inhibited L1. However, this study was designed to focus on the learning process of a grammar rule and not on this particular issue, so we are not in a position to confidently opt for one or the other explanation. For future studies, it would be nice if a second Spanish is run after a break to have a better picture of the story.

### General discussion

Based on both the behavioral results and the electrophysiological responses, we found that when a grammar rule is taught individually in an intensive training paradigm, learning can occur rapidly (within hours) and it is usually accompanied by changes in neural responses that are similar to L1-like patterns, as it has been shown in previous studies (Mueller et al., 2005, 2007, 2008; Davidson and Indefrey, 2009a,b, 2011). Nevertheless, these results differ from other studies finding that adult learners' brain responses differed from the ones of native speakers (Pakulak and Neville, 2011; Meulman et al., 2014; Díaz et al., 2016). We hypothesize that the source of these different results could come from the fact that training studies, because they measure responses earlier in the learning process, may capture different dynamics than longer-term studies. Nevertheless, some other studies that also compared native speakers and adults learners did show native-like patterns in adult learners (Kotz et al., 2008). Moreover, Morgan-Short et al. (2010) suggested that the differences and similarities between natives and learners are not only dependent on the maturity of the learners, but on the interaction of several different factors such as age, proficiency, training type; (see also (Caffarra et al., 2015)). Another interpretation that has been given to these differences is that they could reflect a transitional stage where participants have not reached the proficiency that would lead to an L1-like electrophysiological response (Osterhout et al., 2006). Moreover, Tanner et al. (2014) suggested that the differences found at individual level reflect the stage of L2 acquisition. The present study was designed to study the brain dynamics during the learning at early stages rather than mid- to long-term learning and consolidation. Given this constraint our design does not allow us to reach a strong conclusion about whether learners of Basque would achieve Spanish-like responses in the long term. However, we think that it is important to work in this direction to have a better understanding of why and when discrepancies and similarities between native speakers and adult learners arise. For example, Meulman et al. (2014) showed that learners of Dutch did not show native-like brain responses to gender violations. Díaz et al. (2016) suggest that differences like these are related to language distance. They tested Spanish native speakers who had learned Basque early or late in life, using sentences that could contain a syntactic violation on subject-verb agreement (present in both L1 and L2), object-verb agreement (agreement exists in L1, but this specific agreement is only present in L2) or ergative agreement (unique to L2). They found that regardless of AoA, participants did not show native-like brain responses to the rule only present in L2.

Nonetheless, even though we looked only at “single rule learning,” in our view, the main finding in the present study is the localization of grammar learning. It has been suggested that different subsystems of language rely on different cortical areas that could show different degrees of plasticity (Sanders et al., 2008). Syntax and phonology are known to be the subsystems which present more difficulties when are learned in adulthood. While several studies looked at the source localization of tasks related to phonology learning (for a review see Zhang and Wang, 2007), the literature provides few studies for source reconstruction of tasks that reflect early stages of grammar learning (Davidson and Indefrey, 2009b; Hultén et al., 2014).

Although ultimate attainment is still debated, recent studies have focused on comparison of native speakers vs. non-native speakers that were familiar with the language for a long time prior to the study (Meulman et al., 2015; Díaz et al., 2016; Hanna et al., 2016; Johnson et al., 2016; Sung et al., 2016). However, few of them used a technique that allowed for source localization (Hanna et al., 2016). We think that the present study could contribute to this line of research by showing that it is possible to characterize brain dynamics during grammar learning, and compare the L1 and L2 response within participants. Hopefully, the results presented here could serve as priors for confirmatory studies in this relatively understudied field.

When looking at the training blocks, first of all we see a commonality among the last two blocks: Evoked activity for the control and violation conditions was found in the left hemisphere, more exactly in left temporal (inferior, middle, and superior gyri) and parietal (supramarginal, pre- and post-central gyri) lobes and left angular gyrus (Note: This was not the case for the first training block, see below). There are not many MEG studies that focus on early grammar learning on adults learners, but in this field, (Davidson and Indefrey, 2009b) studied the MEG response to phrase-order violations in German learners of Dutch, and a source reconstruction of that activity implicated a variety of left-hemisphere perisylvian (inferior frontal and left temporal lobe) areas showing a greater amplitude response to grammatical violations months following formal coursework. In addition, Hultén et al. (2014) trained Finnish native speakers to produce short phrases in a miniature artificial language requiring morphosyntactic object agreement. After 4 days of training participants were tested with a MEG production task. Agreement modulated the evoked response strength in left superior temporal and right occipito-temporal cortex. Despite the commonalities just described, we were able also to see some dynamics during the training, in the sense that different areas were more predominantly active in different blocks.

When looking to the evoked activity patterns found on the first training block differ clearly from the patterns found in the other Basque blocks and these later blocks resemble the patterns found in Spanish (L1). More precisely, the source reconstructions show that in the first training block evoked activity is found in the left perisylvian network in both the control and violation conditions. However, in the following Basque blocks the left perisylvian network increased activity is only found in the violation conditions. Although this is the general pattern found in the evoked activity, remember that the statistical support is not supporting the whole pattern, but only some of the areas. As mentioned in the introduction, we present this work as an exploratory study. We are aware that when using an unrestricted analysis the statistical power is diminished, nonetheless, as initial stages of L2 learning remain under-studied we are not in a position to make strong assumptions about the areas involved and restrict our analysis to those. Ideally, the patterns found in this study would help other studies to have a more constrained hypothesis and to perform a more classical confirmatory analysis which would gain statistical power.

In Davidson and Indefrey (2009b), the localization of the effect in the left perisylvian varied depending on the session of the recording (2 weeks or 3 months of formal course in Dutch), where the responses of the early sessions localized the effect at left superior temporal areas, similar to what we report here. Moreover, the first training block violation condition also shows increased evoked activity on the right supramarginal gyrus compared to the control condition, but the evoked activity in these region is not modulated by condition in the other blocks. According to Indefrey (2006) review of the fMRI literature, task effort may be a factor. For example, the differences we found in the early Basque blocks and Spanish block could reflect the degree of effort involved in the task, and the similarities we found between L1 and L2 after the intensive training could reflect that participants learnt to perform the task more effectively. The review of Indefrey (2006) described differences between L1 and L2 BOLD signal found during syntactic processing, especially when a metalinguistic judgment is required during the task. The studies that focused on morpheme inflection showed stronger activation of the dorsal left posterior IFG after training (2 months) compared to before training for L2 morpheme processing (no significant BOLD difference was found), and the area overlaps with the L1 processing area. When the same study was conducted on L2 learners with longer training (6 years) the BOLD signal in this area was much weaker. Indefrey suggests that when an area is involved in a linguistic task, at the beginning greater neural activity of this area could reflect the degree of effort put into the task, while after years of training the weaker activity could reflect that the task has being processed more effectively. Similarly, Zhang and Wang (2007) reviewed several phonetic and tone learning studies, and found that different fMRI studies showed that after some training, improvements in performance were associated with increased BOLD signal in some areas from the left language network. However, after long-term learning, advanced learners, showed decreased BOLD signal after training. They suggested that these patterns reflect that cortical representations can change continuously with learning. We think that the change of evoked activity we found at source level from the first training block to the following blocks is reflecting the progressive changes Indefrey (2006) and Zhang and Wang (2007) suggested. However, again, one cannot exclude intrinsic variability as an explanation for the block-to-block differences seen during training (see Section: Discussion on training blocks). Our goal here is to give an initial description of this variation, so that following studies can investigate whether the patterns are robust across different grammar rules and language combinations.

Finally, as described by Mueller et al. (2007), we also want to emphasize that the materials consisted of a fragment of grammar, and that high level performance in this given task cannot be taken as proof of native-like proficiency in the “complete L2.” It remains to be seen whether individual rule learning is reflected in similar activity when the rule is learned as part of a larger set of rules, or other aspects of the language.

## Conclusion

The behavioral results show that, at least for small fragments of language and simple grammar rules, L2 adult learners can reach a high level of proficiency. We would like to emphasize that it is a miniature language, and that high level performance in this given task cannot be taken as proof of native-like proficiency in L2. More work should be done to understand how L2 learners processes complete' syntactic information.

Furthermore, we have shown that electrophysiological responses during L2 processing that are similar to L1 responses can be seen after some hours of training (despite not being completely equal). Therefore, we conclude that changes in L2 processing can be found in short periods of time and we suggest that models of L2 learning should account for these rapid changes.

## Author contributions

Conceived and designed the experiments: DD, AB. Performed the experiments: AB. Analyzed. the data: AB. Contributed reagents/materials/analysis tools: DD, AB. Wrote/edited the paper: DD, AB.

## Funding

Support for this project was provided to DD by Spanish Ministry of Science and Innovation (MICINN) under the program “Plan Nacional” (grant reference PSI 2011-24802) and to AB by the Basque Government (Eusko Jaurlaritza) under the program “Ikertzaile ez doktoreen doktoretza-aurreko formakuntza-programa” (grant reference PRE_2015_2_0208).

### Conflict of interest statement

The authors declare that the research was conducted in the absence of any commercial or financial relationships that could be construed as a potential conflict of interest.

## Footnotes

^1^Phonological description Levensthein distance mean = 0.77, standard deviation = 1.4 phonemes.
